# Bibliography

**Published:** 1904-11-15

**Authors:** 


### BIBLIOGRAPHY.

A NEW edition of Burchard's "Pathology and Therapeutics" has just come from the press of Lea Bros. & Co. It is a handsome volume of over six hundred pages, and is edited by Dr. Otto E. Inglis, Professor of Pathology and Therapeutics in the Philadelphia Dental College. The work is well illustrated and beautifully printed. It is one of the best made dental text-books, from its mechanical aspect. The form and text has been considerably changed and revised, and brought to conform with our present knowledge of the subjects treated. The materia medica department has been left out, and its matter incorporated in the text under discussion of diseases to which it pertains. This department in the former edition was so brief and unsatisfactory that the book has not suffered by omitting it. As a literary production the work is very well done, but it has lost the beautiful rhetorical flow of language and remarkable clearness of statement which characterized the first edition. Few writers could express themselves as Dr. Burchard, and the present editor has had a difficult and embarrassing task to undertake to revise the work of such a master of expression. There is so much that is good in this work that we are thankful to have accessible, that one hesitates to make criticisms, especially when these are not really things that have been erroneously or unintentially done, but are largely matters of judgment as to the amount and kind of consideration given to certain subjects. As we read the book, it is largely a work on dental pathology, and its therapeutics is made secondary all through the text, except in one instance where some ten pages are given to the subject of destroying pulps with arsenic, which is now almost an obsolete practice. In many other places the therapeutics is unnecessarily complicated with operative details which are fundamental in all such procedures. About one hundred and forty pages are given to embryology and histology, and it is very well done, but it is rather disproportionate to other subjects of more direct interest. Neuralgias and other reflex disorders get only ten pages, and all oral infectious diseases are covered in twelve more pages. If the work fails to accomplish its object, it will be perhaps because it has attempted to do two very important subjects justice within the practicable limits of an ordinary-sized textbook. The two subjects are so closely related that they could be very profitably discussed together, but if this is to be done the next edition should be greatly enlarged and every important topic should have its proportionate space.

